# In Search of Optimal Practice: A Retrospective Comparative Study of Single- Versus Dual-Venous Anastomosis in Microvascular Flaps

**DOI:** 10.7759/cureus.58573

**Published:** 2024-04-18

**Authors:** Deviprasad Sulli, Chandni S., Amar Rao

**Affiliations:** 1 Plastic Surgery, Yenepoya Medical College, Mangalore, IND; 2 Surgical Oncology, Yenepoya Medical College, Mangalore, IND

**Keywords:** single versus two veins, oral cancer reconstruction, venous anastomosis, free flap, microvascular surgery

## Abstract

Background

In the current era of reconstructive surgery, microvascular free flap transfers are the most popular reconstructive procedures performed. The main reason for the failure of the flap and re-exploration is venous thrombosis. Traditionally, most surgeons prefer performing two-vein anastomoses. There is insufficient literature to support that dual-venous anastomosis is better than single-venous anastomosis. In this study, we compared the success rate of single-venous anastomosis with dual-venous anastomosis of different free flap reconstructions.

Methodology

The retrospective cohort study was conducted with a total of 101 patients. Eighty-three were in the one-vein group, and the remaining 18 were in the two-vein group. Outcomes were compared between the two groups regarding re-exploration and flap failure.

Results

The overall success rate of free flap reconstruction was 96%. Among the 101 free flaps, 16 flaps had signs of venous compromise and were re-explored. Out of the 16 flaps, 11 flaps (13.2%) were in the one-vein group, and 5 flaps (27.7%) were in the two-vein group. In the two-vein group, 100% of the flaps were salvaged, and in the one-vein group, only 63% of the flaps were salvaged after re-exploration. There was no statistically significant difference between the one-vein group and the two-vein group concerning re-exploration.

Conclusions

The rate of re-exploration was lower in the one-vein group when compared to the two-vein group. However, this difference was not statistically significant. Hence, a single-vein anastomosis is sufficient for a successful microvascular free flap. However, the rate of flap salvage is better with two-vein anastomosis if there is venous congestion.

## Introduction

In the current era of reconstructive surgery, microvascular flap transfers are the most popular reconstructive procedures performed for various types of defects. In 1989, Hidalgo performed the first free flap for repair and reconstruction of mandibular defects [1]. Since then, several advances in microvascular surgery for free flap transfer have been established to improve the success rate of tissue transfer. The success of free flap surgery is reliant on an effective arterial and venous anastomosis. The main reason for the failure of the flap and re-exploration is venous thrombosis; hence, it is crucial to prevent it, to achieve a better success rate [2-5]. Traditionally, most surgeons prefer performing two-vein anastomoses. There is insufficient literature to support that dual-venous anastomosis is better than single-venous anastomosis. Many studies have shown that dual-venous anastomosis can prevent the incidence of postoperative venous thrombosis in a free flap by a method of self-compensation and offers protection against venous congestion when one of the venous anastomoses is occluded [6-9]. On the contrary, the study conducted by Hanasono et al. proposed that dual anastomoses could reduce the venous flow velocity, causing stasis of blood and predisposing to the development of venous thrombosis [10]. Hence, in this study, we compared the success rate of single-venous anastomosis with dual-venous anastomosis of different free flap reconstructions.

## Materials and methods

The retrospective cohort study was conducted at the Department of Plastic Surgery, Yenepoya Medical College Hospital, Karnataka, India, between 2019 and 2022. A total of 101 patients who underwent microvascular reconstructions after head and neck cancer resection were included in this study.

The patients were divided into two groups depending on the number of venous anastomoses performed during revascularization of the microvascular flaps. Eighty-three (82.17%) patients were in the one-vein group, and the remaining 18 (17.82%) patients were in the two-vein group. All patients of either gender who underwent free flap reconstruction for head and neck malignancy with either one- or two-venous anastomoses were included. Patients who had flap loss after one week were excluded from the study.

The decision for the number of vein anastomoses was made at the surgeon's discretion. All the venous anastomoses were hand-sewn by a single surgeon using an operating microscope or x4 magnification surgical loupes depending on the availability of the microscope. Nylon sutures of size 8-0 and standard microsurgical instruments were used for the anastomosis. All patients received 60 mg of Enoxaparin subcutaneously just before arterial anastomosis. It was continued once a day for five days after surgery.

All the flaps were monitored closely for one week to detect early signs of venous congestion or arterial insufficiency. Flaps were evaluated clinically by color and rate of bleeding on a scratch test. The flap was deemed congested when brisk, dark-colored bleeding was observed from the flap during the scratch test. Patients were immediately transferred to the operation theater, and the flaps were re-explored.

Salvage procedures like the evacuation of hematoma (3, 18.75%) and re-anastomosis (13, 81.25%) were performed as per the findings during re-exploration surgery.

Data about the demography of the patients, diagnosis, type of free flap reconstruction, number of veins, re-exploration, salvage of the flap, and previous radiotherapy history were entered in MS Excel. Analysis was done using IBM SPSS Statistics for Windows, Version 27.0 (IBM Corp., Armonk, NY). Data were presented as frequency and percentage for categorical data, and summary statistics were reported for continuous variables. Chi-square analysis was done to analyze the association between specific variables. Yates' correction was applied when the cells having values less than 5 were more than 20%. The Mann-Whitney U test was applied to compare continuous variables with dichotomous data. A *P*-value < 0.05 was considered to be statistically significant.

## Results

A total of 101 patients who underwent free flap reconstruction for head and neck malignancy were included in the study. The one-vein group comprised 83 (82.17%) free flaps, while the two-vein group included 18 (17.82%) free flaps.

The overall success rate of free flap reconstruction was 96%. The most commonly performed flaps in both groups were the radial forearm free flap (RFFF) and free fibula flap. Other flaps that were performed are the anterolateral thigh flap and medial sural artery perforator flap. The most common oncological procedures done were composite resection and wide local excision for head and neck malignancy. A total of 16 cases required to be re-explored. All were due to venous thrombosis.

The association of age, gender, and preoperative treatment with radiation was analyzed with flap outcome and was noted to have no significant difference in both groups (Table 1). The comparison of the demographic, preoperative radiation therapy, and re-exploration among patients with one-vein and two-vein showed no significant difference (Table 2).

**Table 1:** Correlation of patient demographics and flap outcome.

Variables		Total no. cases (*n* = 101)	Flap outcome		*P*-value
			Survived	Failed	
Age (years)			45.75 ± 9.378	45.25 ± 12.816	0.847
Gender	Male	84 (83.16%)	82 (97.6%)	2 (2.4%)	0.131
	Female	17 (16.83%)	15 (88.2%)	2 (11.8%)	
Preoperative radiation		22 (21.78%)	21 (95.45%)	1 (4.55%)	
No. of veins	One-vein	83 (82.18%)	79 (95.2%)	4 (4.8%)	0.342
	Two-vein	18 (17.82%)	18 (100%)	0	
Re-exploration	Yes	16 (15.84%)	12 (75%)	4 (25%)	<0.001
	No	85 (84.16%)	85 (100%)	0	

**Table 2:** Comparison of flap outcome between single-vein and two-vein groups. RFFF, radial forearm free flap; MSAP, medial sural artery perforator flap; ALT, anterolateral thigh

Variables		One-vein group	Two-vein group	*P*-value
Age (years)		43.62 ± 5.845	44.61 ± 9.041	0.849
Gender, *n* (%)	Male	67 (79.7%)	17 (20.3%)	
	Female	16 (94.1%)	1 (5.9%)	
Preoperative radiotherapy		20 (90.9%) (3 re-explored)	2 (9.1%) (1 re-explored)	0.347
Distribution of the type of flap in both groups, *n* (%)				
RFFF		47 (46.5%)	17 (16.8%)	0.027
Free fibula flap		25 (24.75%)	1 (0.99%)	
MSAP		1 (0.99%)	0	
ALT flap		10 (9.9%)	0	
Re-exploration	Yes	11 (13.2%)	5 (27.7%)	0.155
	No	72 (86.8%)	13 (72.3%)	

Among the 101 free flaps, 16 flaps had signs of venous compromise and were re-explored. Out of the 16 flaps, 11 flaps (13.2%) were in the one-vein group and 5 flaps (27.7%) were in the two-vein group. In the two-vein group, 100% of the flaps were salvaged, while in the one-vein group, only 63% of the flaps were salvaged.

## Discussion

Free tissue transfer with microvascular anastomosis has emerged as a cornerstone procedure for the reconstruction of complex surgical defects, particularly following oncological procedures. A comprehensive meta-analysis conducted by Riot et al. reported an impressive success rate of 97.48% for free flaps, underscoring the efficacy and widespread adoption of this technique [7]. The local factors that intricately influence the success rate of free flaps include pedicle length, the vascular anastomotic technique, the position of the vascular pedicle, the placement of the drain, and the immobilization of the head [11-13]. However, thrombosis of anastomotic vessels remains the most common and significant complication, accounting for 61.7% of cases requiring re-exploration [14]. Vascular compromise typically manifests within the initial 24 hours post-surgery, highlighting the criticality of vigilant postoperative monitoring [15].

The most important factors in the pathogenesis of thrombosis are blood flow velocity across the anastomotic site and the endothelial injury. The exposed subendothelium leads to the activation of platelets [16-19]. To prevent the thrombogenic situation, the blood flow through the anastomotic site should be at a rate higher than the thrombogenic threshold [18,19]. If the blood flow through the pedicle vessels is sluggish, it is associated with a significant increase in the risk of thrombus formation, as shown in several clinical and experimental studies [10,19].

There has always been a debate about whether dual-venous anastomosis is superior to single-venous anastomosis to avoid venous complications in free flaps. It was proposed by Enajat et al. and Ross et al. that dual-venous anastomosis could increase the number of venous flow routes and thus prevent thrombosis [8-9]. On the other hand, Hanasono et al. argued that dual-venous anastomosis would reduce the blood flow velocity and cause stasis, leading to the development of venous thrombosis [10]. Futran and Stack proposed that the success rate of single- versus dual-venous anastomosis is indifferent and that dual-venous anastomosis prolonged the surgical time [5].

In our study, we noted 16 cases of postoperative congestion, which required re-exploration. The percentage of flap re-exploration was noted to be higher in the dual-venous group (27.7%) compared to the single-venous group (13.2%). The higher re-exploration rate in the dual-venous group may be attributed to the increased probability of thrombosis, as suggested by Hanasono et al. in their study. They measured the velocity of blood in venae comitantes before pedicle division and after the completion of the anastomosis, concluding that single-venous anastomosis has a positive effect on flap survival and helps prevent venous thrombosis [10]. However, this difference was not statistically significant. A retrospective study by Boczar et al. also concluded that there is no statistically significant difference in venous congestion between one-vein and two-vein groups [20].

Although there was a higher exploration rate in the dual-venous group, all the flaps were salvaged in the dual-venous group compared to the single-venous group. A recent meta-analysis by Matthews et al. also concluded that dual-venous anastomosis reduces the rate of re-exploration due to venous congestion [21].

Additionally, the impact of preoperative radiation therapy was considered as one of the factors for flap failure. In our study, 22 patients had preoperative radiation, but only one flap failure was noted. This finding is consistent with a study conducted by Bourget et al., which found no negative effects of radiation on free tissue transfer survival [22].

While our study offers valuable insights, it is not devoid of limitations, including its retrospective nature, uneven case distribution, and single-center design. Future research endeavors should address these constraints to further refine our understanding and enhance clinical outcomes in free tissue transfer.

## Conclusions

Free tissue transfer with microvascular anastomosis represents a pivotal advancement in reconstructive surgery. Despite the high success rate, the occurrence of thrombosis remains a significant challenge. The debate surrounding single- versus dual-venous anastomosis persists, with our study contributing valuable insights into the comparative outcomes. Our findings indicate no statistically significant difference in outcome between one vein or two vein groups. Even with a higher rate of re-exploration in the two vein groups, all flaps were salvaged. A single-venous anastomosis is sufficient for a successful microvascular free flap. However, the chance of flap salvage is higher in the two-vein group if there is venous congestion.

For a more definitive conclusion on the effectiveness of single- versus dual-venous anastomosis, a larger, prospective, and multicenter study with equal group sizes would be needed.
